# The Influence of Preoperative Physical Activity on Intraoperative Brain Function in Cardiac Surgical patients

**DOI:** 10.21203/rs.3.rs-4427122/v1

**Published:** 2024-06-07

**Authors:** Rushil Vladimir Ramachandran, Ajay Ananthakrishnan, Hibiki Orui, Kestutis Kveraga, Balachundhar Subramaniam

**Affiliations:** Beth Israel Deaconess Medical Center; Beth Israel Deaconess Medical Center; Beth Israel Deaconess Medical Center; Beth Israel Deaconess Medical Center; Beth Israel Deaconess Medical Center

**Keywords:** cardiac surgery, delirium, anesthesia, pre-operative physical activity, preventative health

## Abstract

**Background:**

Preoperative physical activity and intraoperative brain health are recognized to influence postoperative delirium (POD). Electroencephalogram (EEG) burst suppression and cerebral desaturation are indicators of abnormal intraoperative brain health. Our study aimed to investigate the associations between preoperative physical activity and intraoperative EEG burst suppression and cerebral desaturation.

**Methods:**

We retrospectively analyzed data from 67 patients from one of the institutions participating in a multisite randomized controlled trial, PANDORA, involving patients undergoing cardiac surgery. The preoperative PCS12 score calculated using the SF12 questionnaire was used as an indicator of preoperative physical activity. Intraoperative EEG and cerebral oximetry data (not the current standard of care in this facility) were collected, and the anesthesiologists were blinded to the information. We analyzed the following associations between the PCS12 score and i) burst suppression duration, ii) the number of cerebral desaturations, and iii) the number of observations with concurrent cerebral desaturation and burst suppression using a generalized linear model. The results are presented as percentage changes in outcomes, and a 95% C.I. p value < 0.05 was considered to indicate statistical significance.

**Results:**

Each unit increase in the PCS12 score was associated with a 3.3% decrease in the duration of burst suppression (−3.3 [−5.3, −1.2], p value = 0.002). The duration of burst suppression decreased by 29.2% with each successive quartile increase in the PCS-12 score, indicating a dose−response relationship (−29.2 [−41.6, −16], p < 0.001). Specifically, the patients in the last three quartiles exhibited a 55.4% reduction in BSD compared to those in the first quartile (−55.4 [−74.4, −24.6], p = 0.002) (Fig. 2). We did not observe any significant association between the PCS12 score and cerebral desaturation.

**Conclusion:**

Decreased preoperative physical activity, as measured by the SF-12 questionnaire, is significantly associated with increased EEG burst suppression duration. Preoperative physical activity did not show any association with cerebral desaturations and concurrent cerebral desaturation and burst suppression.

**Clinical Trial information:**

ClinicalTrials.gov Identifier- NCT04093219

https://clinicaltrials.gov/ct2/show/NCT04093219

Principal Investigator - Balachundhar Subramaniam

Date of registration - September 13, 2019

## Background

Postoperative neurocognitive disorders such as postoperative delirium (POD) are common after cardiac surgery and have a detrimental impact on patients’ health and well-being^1^. Elderly patients are particularly susceptible to developing such disorders, leading to long-term cognitive decline, decreased self-dependency, and increased morbidity and mortality following surgery. The etiology of POD in cardiac surgical patients is complex and can be attributed to numerous intraoperative factors, such as inflammation from surgical insult and neuronal or vascular damage from surgical complications, cardiopulmonary bypass (CPB), or anesthesia exposure^2^. Preoperative risk factors, such as age, preexisting dementia, and diabetes, further complicate this etiology^1^.

Older patients are also more likely to be frail and deconditioned and have limited physical activity before surgery due to the physical constraints of their disease^3^. Previous research has shown that exercise capacity and physical function are predictors of mortality and morbidity after cardiac surgery^4^. Low exercise capacity is also associated with a greater incidence of POD^5^. However, its impact on intraoperative brain health remains unexplored. Intraoperative brain health is a significant predictor of POD and can be monitored through electroencephalography (EEG) and regional cerebral oxygen saturation (rSO2). Intraoperative EEG Burst suppression (BS), defined as periods of marked suppression of brain electrical activity alternating with bursts of activity and cerebral desaturation, is characteristic of poor intraoperative brain health and is believed to contribute to POD^2, 6, 7^. In our recent work (unpublished), observations of burst suppression were significantly concurrent with observations of cerebral desaturation, indicating a possible link between cerebral desaturation and burst suppression. This finding also indicates the possibility that the cerebral desaturations could have led to burst suppression. Hence, we propose concurrent cerebral desaturation and burst suppression as additional indicators of poor intraoperative brain health. Exploring the link between diminished preoperative physical activity, which is common among cardiac surgery patients, and intraoperative brain health will allow anesthesiologists to better predict and manage elderly patients’ health intraoperatively and prevent POD.

The association between preoperative frailty and intraoperative brain health has been previously explored in noncardiac surgery patient populations. Boncompte et al. studied whether intraoperative alpha power predicts preoperative frailty and found no association^8^. Another study of elderly noncardiac surgery patients revealed that preoperative frailty was associated with increased intraoperative cerebral desaturation episodes^9^. The association between preoperative physical activity and intraoperative brain health in cardiac surgeries has not been extensively explored. We primarily hypothesized that low preoperative physical activity would be associated with an increased duration of intraoperative BS, cerebral desaturation, and concurrent cerebral desaturation and burst suppression. Additionally, we aimed to explore the association between preoperative physical activity and length of stay in the hospital and the ICU.

## Methods

The study participants were cardiac surgical patients aged > 60 years who required CPB and who were undergoing coronary artery bypass graft and/or valve repair. Patients who underwent aortic surgeries were excluded. This study is a retrospective analysis of prospectively collected blinded EEG and cerebral oximetry data from a single site. Cerebral oximetry was measured using near-infrared spectroscopy (NIRS). Both EEG and cerebral oximetry monitoring were started in the preoperative waiting room with the application of an EEG and an NIRS sensor on the participants’ foreheads connected to a single monitor, and the data were continuously collected until the end of surgery. The EEG sensor has four leads, providing four simultaneous channels of frontal EEG waveforms. SEDline monitors and sensors, sponsored by their manufacturer Masimo Incwere used to collect this data. The clinicians were blinded to the data; hence, these data were not used for patient management during surgery. Typically, at our institution during the study period, anesthesia was induced with intravenous fentanyl and propofol or etomidate (based on patient characteristics and anesthesiologist preference), and rocuronium was given for skeletal muscle relaxation. Anesthesia was maintained with 0.5–1.0% isoflurane in 100% oxygen with supplemental intravenous fentanyl given as intraoperative analgesia. Patients were ventilated with a tidal volume of 6 ml/kg, and respiratory rates were adjusted to maintain a pCO2 of 40–55 mmHg and a pH greater than 7.25. Patients were placed on a nonpulsatile CPB pump using arterial and venous cannulas. The perfusionist titrated the CPB flow to achieve a venous saturation of > 60% and a mean arterial pressure (MAP) of 50–70 mm Hg unless otherwise specified in discussion with the attending anesthesiologist and surgeon. Intraoperative brain monitoring is not the current standard of care in our facility.

Abnormal intraoperative brain health was assessed using the primary outcome, duration of BS, and secondary outcomes, number of observations of cerebral desaturation and concurrent cerebral desaturation and burst suppression, respectively. BS is defined by marked suppression of brain electrical activity alternating with bursts of activity. BS duration (in minutes) was calculated per patient from the raw EEG data from the SedLine monitor, using a recursive variance algorithm adapted from Westover et al^11^. Burst suppression was calculated as a percentage of time for each minute of surgery that EEG activity fell below-defined variance criteria bounds, using the mean of raw, preprocessed EEG of the four channels acquired by the Masimo Sedline monitor. The automated burst suppression detection was verified via visual spot checks by an experienced cognitive neuroscientist. Regional rSO2 measurements were recorded as real-time observations (every 2 seconds) from the right and left cerebral hemispheres. Cerebral desaturation was defined as an observation with an RSO2 < 60%^12^, and we calculated the number of cerebral desaturations (2-second observations) per patient. Apart from the raw EEG, the monitor also calculated the processed EEG index, the suppression ratio (SR), from the raw EEG recording. The monitor calculated this ratio as the percentage of EEG recordings showing burst suppression during the previous minute, which was refreshed every 2 seconds. The SR values were represented as whole integers from 0–100%. An observation showing an SR of 1% indicates 0.6 seconds (1% of a minute) of burst suppression in the last minute and is the lowest discrete incidence of burst suppression the monitor captures. Simultaneous observations of the SR and cerebral saturation (2-second intervals) were compared for analysis of their concurrence. Concurrent cerebral desaturation and burst suppression were defined by simultaneous observations of SR > 0 and cerebral desaturation. We calculated the number of observations of concurrent cerebral desaturation and burst suppression per patient (number of 2-second interval observations).

The SF-12 is a questionnaire that assesses the impact of health on everyday quality of life^13^. A study team member administered the questionnaire to all patients either remotely or in person before their surgery. The SF-12 has twelve questions measuring eight health domains. The first six questions measure physical health (PCS12), and the last six questions measure mental health (MCS12). The four physical health-related domains are the following: general health, physical functioning, role physical (limitations in usual role activities due to physical health), and bodily pain. The four mental health-related domains are the following: vitality, social functioning, role-emotional (limitations in usual role activities due to emotional problems), and mental health. The PCS12 score was computed for each patient from the responses to questions assessing the physical health-related domains (the first six questions). The United States population’s average normalized PCS12 score is 50 ± 10. The PCS12 has been evaluated on cardiac surgical patients in previous studies. One study looking at 163 patients aged 80 years or older at the time of surgery for aortic stenosis had a baseline score of 44.68 (~ .5 SDs below US population average); another analysis studying a similar population of 628 patients found a baseline PCS score of 30.2 (~ 2 SDs below US population average)^14, 15^.

The PCS12 score was used as an indicator of the patient’s preoperative physical activity. Data on exploratory outcomes such as hospital length of stay (HLOS) and intensive care unit length of stay (ICULOS) were extracted from the hospital’s online medical records.

### Statistical analysis:

An a priori sample size calculation was not conducted, and a convenience sample was utilized. We analyzed the following associations: PCS12 scores and i) burst suppression duration (calculated manually using a minimum variance algorithm from raw EEG by the neuroscience expert), ii) number of cerebral desaturations (calculated from the rSO2 values from the monitor), and iii) number of observations with concurrent cerebral desaturation and burst suppression (calculated from the SR and rSO2 values from the monitor). Owing to the continuous non normal (right-skewed) distribution of the outcomes, a generalized linear model using the gamma distribution and log link function was used. Previous literature suggests an association between age and gender and between age and burst suppression^14^. Additionally, the duration of intraoperative EEG and rSO2 monitoring influences the duration of burst suppression and cerebral desaturation. Therefore, we included the duration of brain health monitoring, age, and sex as covariates. We also analyzed the associations between the PCS12 score and the HLOS and ICU-LOS using Kendall’s rank correlation. The results are presented as percentage changes in outcomes with unit changes in the predictors, with 95% confidence intervals. All the statistical analyses were performed using R version 4.2.2. P < 0.05 was considered to indicate statistical significance.

## Results

### Baseline characteristics (Table 1)

Enrollment of participants in the PANDORA clinical trial began in the fall of 2020. The patients’ baseline characteristics are presented in Table 1. The data of 91 participants were analyzed in this study, and none of the participants experienced delirium at the baseline neurocognitive assessment. Of the 91 participants, 67 were included in the analysis based on the continuous availability of rSO2 and BS data for the entire duration of surgery.

### PCS-12 and intraoperative burst suppression

**Figure 1:**
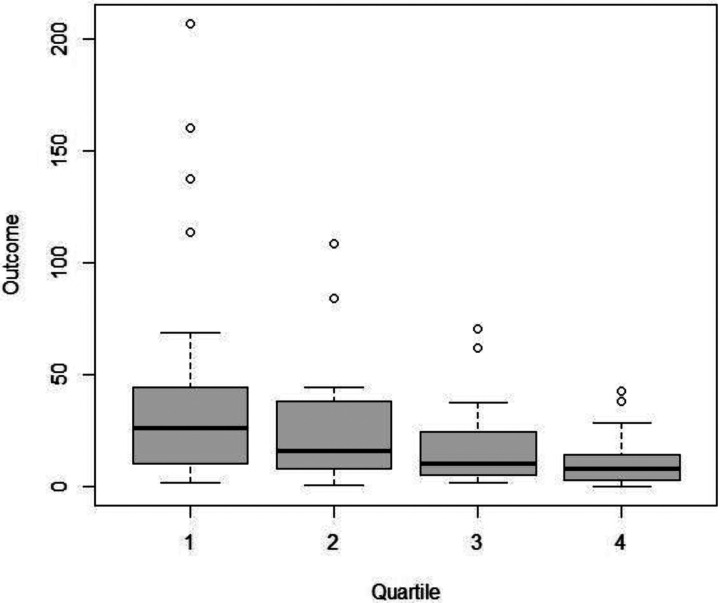
PCS12 quartiles and BSD

**Figure 2:**
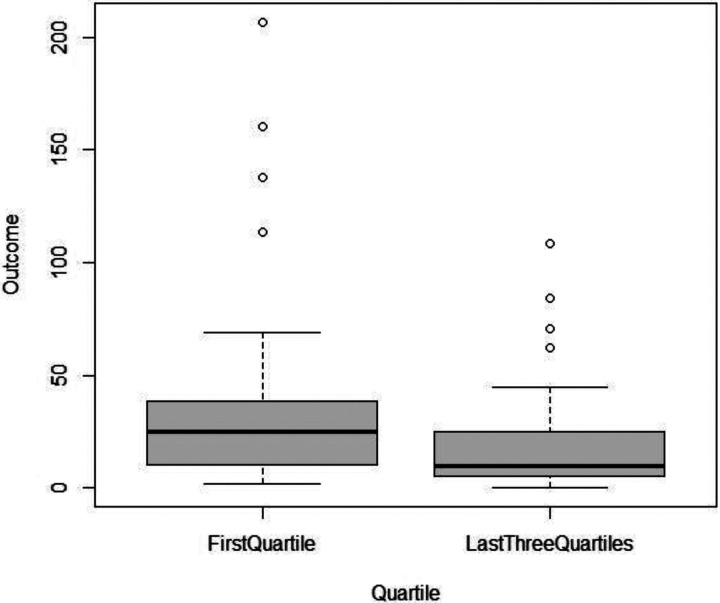
BSD for the first and combined last three PCS12 quartiles

Each unit increase in the PCS12 score was associated with a 3.3% decrease in the duration of burst suppression (−3.3 [−5.3, −1.2], p value = 0.002) (Table 2). Age and sex did not show any association with burst suppression duration. The patients were grouped into quartiles based on the PCS-12 score. The duration of burst suppression decreased by 29.2% with each successive quartile increase in the PCS-12 score, indicating a dose‒response relationship (−29.2 [−41.6, −16], p < 0.001) (Fig. 1 and Table 3). Specifically, the patients in the last three quartiles exhibited a 55.4% reduction in BSD compared to those in the first quartile (−55.4 [−74.4, −24.6], p = 0.002) (Fig. 2).

### PCS-12 and cerebral desaturations (Tables 4 and 5)

We did not observe any significant associations between PCS-12 scores and cerebral desaturations or between PCS12 and concurrent cerebral desaturation and burst suppression.

### SF-12 and HLOS/ICU-LOS

PCS-12 scores did not significantly correlate with HLOS (correlation coefficient = −0. 012, p value = 0.83) or ICU-LOS (correlation coefficient = −0.007, p value = 0.90).

## Discussion

This study is the first to explore the associations between preoperative physical activity and total intraoperative BSD and cerebral desaturation in a cardiac surgery population. This study has two significant findings. First, we observed a significant association between PCS-12 scores and intraoperative BSD. Second, we did not find any significant association between PCS-12 scores and intraoperative cerebral desaturations.

### PCS-12 and BSD:

Our study showed that patients with lower preoperative PCS-12 scores had greater intraoperative BSD. We chose PCS-12 as it is a commonly done measure that is scalable to different patients in different settings including cardiac surgery preoperative evaluation. The literature indicates a positive correlation between physical activity and improved overall brain health, including heightened cerebral perfusion, improved cognitive function, and a reduced prevalence of dementia and POD^15^. In a study of 159 elderly patients (> 60 yrs.) in 2020, Pedemonte et al. reported that low preoperative physical activity scores, as measured by questionnaires, were associated with diminished frontal alpha EEG power, increased odds of BS during the CPB period, and increased odds of developing POD^16^. However, the authors selected a 2-minute EEG segment that represented the maintenance phase of general anesthesia during surgery to identify BS. Although this helps avoid the confounding effects of other factors on burst suppression, the total intraoperative BSD is more clinically meaningful than the odds of burst suppression during a 2-minute segment of the maintenance phase. The results of our study support these previous studies’ hypotheses that increased preoperative physical activity is associated with reduced burst suppression. Additionally, the BSD decreased significantly with each quartile increase in the PCS12 score, indicating a dose‒response relationship.

It is of clinical utility to propose an objective cutoff level of physical activity at which a patient is significantly more likely to have BS. However, the difficulty of objectively measuring preoperative physical activity levels makes such a task difficult. Self-report questionnaires are short and can be conveniently administered to patients in the preoperative setting but they do not independently measure physical activity or fitness. However, in our analysis, patients who scored in the lowest quartile of PCS-12 scores had a greater BSR than did those who scored in the upper 3 quartiles. Therefore, we chose to propose a PCS-12 score < = 41, which was the median PCS-12 score of quartile 2, as a cutoff for a significantly increased risk of BS. Such an assessment can be made more robust by incorporating age, FRAILTY scores, and other preoperative factors to more accurately predict patients at high risk of poor intraoperative brain health.

### PCS-12 score and cerebral desaturation/concurrent abnormal events:

Our study did not find any association between preoperative PCS-12 scores and either intraoperative cerebral desaturation or concurrent abnormal events. To our knowledge, there are no previous studies analyzing physical activity and intraoperative CO. However, previous studies have shown that both low preoperative and intraoperative rSO2 are linked to the occurrence of POD and other adverse events, such as stroke^2^.

Physical activity improves cardiorespiratory fitness and enhances cerebral blood flow in healthy adults. The effect of preoperative physical activity on intraoperative cerebral oxygen saturation has not been evaluated to the best of our knowledge. A previous study aiming to investigate the role of resting cerebral saturation as a risk factor for postsurgical mortality in heart failure patients revealed that decreased preoperative rSO2 at rest was associated with low physical activity^17^. This small study measured preoperative rSO2 and physical activity. Adequately powered studies analyzing the association between preoperative physical activity and intraoperative cerebral desaturation is needed.

### PCS-12 and HLOS/ICU-LOS

The PCS-12 score did not show any association with the HLOS or ICU-LOS. Several studies of cardiac surgery patients have shown that decreased preoperative physical activity, as measured by questionnaires, is predictive of increased length of hospital stay^4, 18^. Additionally, another study showed that increased physical activity in the 5 days following surgery was associated with a decreased length of hospital stay^19^. This is contrary to our study but may be due to the differences in the methods of assessing physical activity.

Our study has limitations. No causal inferences can be made based on these analyses. A convenience sample size was used with no formal sample size estimation due to lack of prior data. The PCS component of the SF-12 measures the effect of physical health on daily quality of life, which we used as a proxy for preoperative physical activity. Past studies have used other questionnaires to measure preoperative physical activity, such as the Corpus Christi Heart Project and Physical Activity Scale for the Elderly (PASE) questionnaires^4^. Given the wide array of subjective questionnaires that measure preoperative physical activity, it is difficult to compare preoperative physical activity across different questionnaires. Objective measures of preoperative physical activity, such as step counts from wearables, are feasible and objective^20, 21^. Limitations in intraoperative data collection included periods of interrupted data collection, lost data, and high noise in certain intraoperative intervals. Those periods were excluded from the analysis. The missing data and the possibility of residual confounding introduce the potential for bias.

## Conclusions

Lower preoperative PCS-12 scores in cardiac surgical patients, indicative of reduced physical activity, correlated with increased intraoperative EEG burst suppression but were not associated with intraoperative cerebral desaturation episodes. These findings highlight the potential for implementing a simple preoperative physical activity assessment to identify patients at risk for intraoperative burst suppression, a predictor of postoperative delirium. Future efforts should be directed to study the effect of improving preoperative physical activity as part of surgical prehabilitation on postoperative cognition.

## Figures and Tables

**Table 1:** Demographics

Demographics	N = 91^1^
**Age**	70 (65, 74)
**Gender**	
Female	27 (30%)
Male	64 (70%)
**Race**	
White	84 (92%)
Black or African Amercian	3 (3.3%)
Asian	2 (2.2%)
More than one race	1 (1.1%)
Other	1 (1.1%)
**Ethnicity**	
Hispanic or Latino	2 (2.2%)
Not Hispanic or Latino	89 (97.0%)
**BMI**	27.8 (24.9, 30.8)
**PCS12**	45 (36, 55)
**BSD (min)**	12 (6, 30)
**Cerebral desaturations (min)**	100 (23, 172)
**Concurrent cerebral desaturation and burst suppression (min)**	10.8 (1, 45)
**HLOS (days)**	6.5 (5.3, 8.6)
**ICU LOS (hours)**	47 (27, 74)
**MoCA score**	25.0 (22.0, 27.0)
**STS risk score (%)**	7 (5, 10)

1 n (%); Median (IQR)

**Table 2:** Adjusted model showing the association between PCS12 and BSD

Characteristic	Percentage change	95% CI	p value
PCS 12 [each unit increase in the score]	−3.3%	−5.3%, −1.2%	0.002
Duration of EEG recording [minutes]	0.3%	0%, 0.6%	0.042
Males [vs Females]	−5.7%	1%, 7.1%	0.066
Age [years]	3.4%	−44%, 54%	0.815

**Table 3:** Adjusted model showing the association between PCS12 score-based quartiles and BSD

Characteristic	Percentage change	95% CI	p value
Quartile	−29.2%	−41.6%, −16%	<0.001
Duration of EEG recording [minutes]	0.2%	0%, 0.6%	0.051
Males [Females]	−4.5%	−43.3%, 53%	0.848
Age [years]	3.3%	0%, 6.8%	0.07

## Data Availability

The datasets used and/or analyzed during the current study are available from the corresponding author on reasonable request.

## Abbreviations

EEG: Electroencephalogram
p-EEG: processed Electroencephalogram
PANDORA: Prophylactic 6- hourly intravenous AcetaminopheN to prevent postoperative Delirium in Older caRdiac surgicAl patients
CPB: Cardiopulmonary bypass

## References

[1] Chen H, Mo L, Hu H, Ou Y, Luo J. Risk factors of postoperative delirium after cardiac surgery: a meta-analysis. J Cardiothorac Surg. 2021;16(1):113. doi:10.1186/s13019-021-01496-w 33902644 PMC8072735

[2] Milne B, Gilbey T, Gautel L, Kunst G. Neuromonitoring and Neurocognitive Outcomes in Cardiac Surgery: A Narrative Review. J Cardiothorac Vasc Anesth. 2022;36(7):2098–2113. doi:10.1053/j.jvca.2021.07.029 34420812

[3] Lee DH, Buth KJ, Martin BJ, Yip AM, Hirsch GM. Frail Patients Are at Increased Risk for Mortality and Prolonged Institutional Care After Cardiac Surgery. Circulation. 2010;121(8):973–978. doi:10.1161/CIRCULATIONAHA.108.841437 20159833

[4] Kehler DS, Stammers AN, Tangri N, Systematic review of preoperative physical activity and its impact on postcardiac surgical outcomes. BMJ Open. 2017;7(8):e015712. doi:10.1136/bmjopen-2016-015712 PMC5724229 28801404

[5] Masato O, Izawa KP, Seimi SK, Preoperative exercise capacity is associated with the prevalence of postoperative delirium in elective cardiac surgery. Aging Clin Exp Res. 2018;30(1):27–34. doi:10.1007/s40520-017-0736-5 28243862

[6] Soehle M, Dittmann A, Ellerkmann RK, Baumgarten G, Putensen C, Guenther U. Intraoperative burst suppression is associated with postoperative delirium following cardiac surgery: a prospective, observational study. BMC Anesthesiol. 2015;15(1):61. doi:10.1186/s12871-015-0051-7 25928189 PMC4419445

[7] Lopez MG, Pandharipande P, Morse J, Intraoperative cerebral oxygenation, oxidative injury, and delirium following cardiac surgery. Free Radic Biol Med. 2017;103:192–198. doi:10.1016/j.freeradbiomed.2016.12.039 28039082 PMC5258679

[8] Boncompte G, Sun H, Elgueta MF, Intraoperative electroencephalographic marker of preoperative frailty: A prospective cohort study. J Clin Anesth. 2023;86:111069. doi:10.1016/j.jclinane.2023.111069 36738630 PMC10074446

[9] Khan SA, Chua HW, Hirubalan P, Karthekeyan RB, Kothandan H. Association between frailty, cerebral oxygenation and adverse post-operative outcomes in elderly patients undergoing non-cardiac surgery: An observational pilot study. Indian J Anaesth. 2016;60(2):102–107. doi:10.4103/0019-5049.176278 27013748 PMC4787120

[10] Khera T, Mathur PA, Banner-Goodspeed VM, Scheduled Prophylactic 6-Hourly IV AcetaminopheN to Prevent Postoperative Delirium in Older CaRdiac SurgicAl Patients (PANDORA): protocol for a multicentre randomised controlled trial. BMJ Open. 2021;11(3):e044346. doi:10.1136/bmjopen-2020-044346 PMC7949372 33692183

[11] Westover MB, Shafi MM, Ching S, Real-time segmentation of burst suppression patterns in critical care EEG monitoring. J Neurosci Methods. 2013;219(1):131–141. doi:10.1016/j.jneumeth.2013.07.003 23891828 PMC3939433

[12] Vretzakis G, Georgopoulou S, Stamoulis K, Cerebral oximetry in cardiac anesthesia. J Thorac Dis. 2014;6(Suppl 1):S60–S69. doi:10.3978/j.issn.2072-1439.2013.10.22 24672700 PMC3966165

[13] Ware JE, Kosinski M, Keller SD. A 12-Item Short-Form Health Survey: Construction of Scales and Preliminary Tests of Reliability and Validity. Med Care. 1996;34(3):220. 8628042 10.1097/00005650-199603000-00003

[14] Jansen Klomp WW, Nierich AP, Peelen LM, Survival and quality of life after surgical aortic valve replacement in octogenarians. Journal of Cardiothoracic Surgery. 2016;11(1):38. doi:10.1186/s13019-016-0432-0 26992390 PMC4799630

[15] Reynolds MR, Magnuson EA, Wang K, Health-Related Quality of Life After Transcatheter or Surgical Aortic Valve Replacement in High-Risk Patients With Severe Aortic Stenosis: Results From the PARTNER (Placement of AoRTic TraNscathetER Valve) Trial (Cohort A). Journal of the American College of Cardiology. 2012;60(6):548–558. doi:10.1016/j.jacc.2012.03.075 22818074

[16] Besch G, Liu N, Samain E, Occurrence of and risk factors for electroencephalogram burst suppression during propofol–remifentanil anaesthesia. Br J Anaesth. 2011;107(5):749–756. doi:10.1093/bja/aer235 21828343

[17] Salzman T, Dupuy O, Fraser SA. Effects of Cardiorespiratory Fitness on Cerebral Oxygenation in Healthy Adults: A Systematic Review. Front Physiol. 2022;13:838450. doi:10.3389/fphys.2022.838450 35309063 PMC8931490

[18] Pedemonte JC, Plummer GS, Chamadia S, Electroencephalogram Burst-suppression during Cardiopulmonary Bypass in Elderly Patients Mediates Postoperative Delirium. Anesthesiology. 2020;133(2):280–292. doi:10.1097/ALN.0000000000003328 32349072 PMC7365754

[19] Zaghi A, Holm H, Korduner J, Cerebral saturation is associated with physical activity and post-discharge mortality in heart failure patients. Eur Heart J. 2022;43(Supplement_2):ehac544.947. doi:10.1093/eurheartj/ehac544.947

[20] van Laar C, TImman ST, Noyez L. Decreased physical activity is a predictor for a complicated recovery post cardiac surgery. Health Qual Life Outcomes. 2017;15:5. doi:10.1186/s12955-016-0576-6 28069013 PMC5220604

[21] Mungovan SF, Singh P, Gass GC, Smart NA, Hirschhorn AD. Effect of physical activity in the first five days after cardiac surgery. J Rehabil Med. 2017;49(1):71–77. doi:10.2340/16501977-2165 28101566

[22] Vasankari S, Hartikainen J, Vasankari V, Objectively measured preoperative physical activity and sedentary behaviour among Finnish patients scheduled for elective cardiac procedures: baseline results from randomized controlled trial. BMC Sports Sci Med Rehabil. 2022;14(1):130. doi:10.1186/s13102-022-00522-1 35842711 PMC9287962

[23] Cook DJ, Thompson JE, Prinsen SK, Dearani JA, Deschamps C. Functional Recovery in the Elderly After Major Surgery: Assessment of Mobility Recovery Using Wireless Technology. Ann Thorac Surg. 2013;96(3):1057–1061. doi:10.1016/j.athoracsur.2013.05.092 23992697

